# Progress in understanding drought tolerance: from alleles to cropping systems

**DOI:** 10.1093/jxb/ery187

**Published:** 2018-06-06

**Authors:** Rajeev K Varshney, Roberto Tuberosa, Francois Tardieu

**Affiliations:** 1 Center of Excellence in Genomics & Systems Biology (CEGSB), International Crops Research Institute for the Semi-Arid Tropics (ICRISAT), Hyderabad, India; 2 Department of Agricultural and Food Sciences, University of Bologna, Italy; 3 Université de Montpellier, INRA, LEPSE, Montpellier, France

**Keywords:** Abortion, crop yield, dehydration, drought tolerance, osmotic adjustment, root architecture, seed development, turgor management

**Improving crop yields under rainfed environments is key to meeting the food security demands of an ever-increasing population, but climate change-associated expansion of drought-affected arable land means that resilient crops and agronomic practices are critical. High-throughput plant phenomics and modern genetic approaches must be directed towards precise understanding of factors controlling crop yield. This special issue covers root dynamics, turgor management under desiccation, molecular responses to dehydration, impact of drought on plant development and seed abortion, and adjustment of traits to the most frequent patterns of drought. It also addresses interdisciplinary views for enhancing genetic gains and achieving a more sustainable climate-resilient agronomy.**

Drought is a devastating factor for global agronomic production. Although it is uncertain whether episodes of drought will be more severe with climate change (Sheffield *et al.*, 2012), their prevalence and year-to-year variability are major features in future modelling scenarios (Sun *et al.*, 2012; Harrison *et al.*, 2014; see also Box 1). Understanding the mechanisms underpinning plant behaviour under drought is a challenge due to differences in (i) the traits that control plant water status under rapidly changing soil water availability and evaporative demand, (ii) the response of plants to changes in water status, its genetic variability and differences between species (e.g. between cereals and legumes), and (iii) interactions with other factors such as the duration and position of the crop cycle in the season, the frequency of episodes with high temperature and the soil chemical composition (e.g. soil salinity or presence of Al).

**Box 1. New climate-resilient crops and agronomic practices**

High temperatures and water deficits associated with climate change are projected to become increasingly erratic, resulting in an expansion of drought-affected arable land worldwide. In such environments, increasing or maintaining crop yields will become gradually more difficult due to the decreasing availability of irrigation water. Therefore, the adoption of climate-resilient crops and agronomic practices is critically important. It is essential to design cropping systems and genotypes that can jointly overcome limitations of crops yields under well-identified drought scenarios. For instance, sorghum is usually grown at high density (as shown). Canopies with half the plant density, obtained by skipping one row out of two (reproducing the crop-free path in the image every second row), reduce canopy cover and offer the advantage of delaying soil water depletion at the end of the cropping season, thereby reducing the risk of failure in very dry years, although it reduces yield in milder years (Whish *et al.*, 2005). This technique may require genotypes with different shoot and root architecture and phenology compared with those used in pure stands.

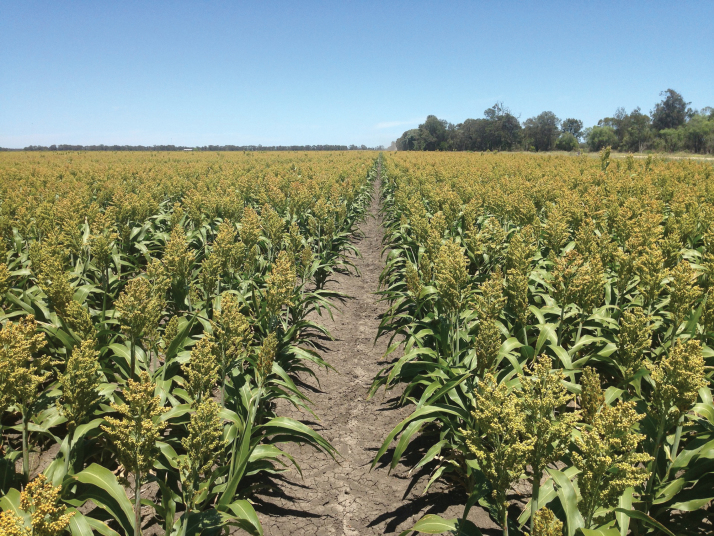

Over past decades, crop physiologists have increasingly focused on molecular aspects of stress tolerance while intensive research and breeding have allowed the selection of climate-resilient cultivars with improved yields. Drought research has progressed rapidly, as can be seen in successive InterDrought conferences (Belhassen, 1997; Araus *et al.*, 2007; Blum, 2013; Tuberosa *et al.*, 2014; see also Tardieu *et al.*, 2017) (see also Box 2, a tribute to Abraham Blum). While initially focused on identifying tolerant lines and understanding the role of proxy traits, research has now reached a position where it can also uncover and allow the manipulation of genes/QTLs and mechanisms involved in cellular and whole-plant responses related to acclimation to drought scenarios. Several loci and genes have been characterized to develop the concept and models of complex drought tolerance traits (Tricker *et al.*, 2018). This paves the way to mapping and cloning of genetic loci governing drought tolerance through turgor management and hormonal regulation, plant architecture and seed development biology, corroborated by transcriptional and post-transcriptional response networks. Drought tolerance involves cell-to-cell to whole-plant level hydraulic or metabolic readjustment, and hormone signalling able to control growth under water deficit. Now, researchers are able to debate the interactions between roots, shoots, the rhizosphere and reproductive traits and how they collectively affect crop yield under the diversity of climatic scenarios involving limited water availability (Tardieu *et al.*, 2018).

**Box 2. A tribute to Abraham Blum (1934-2018)**

Abraham Blum passed away on 10 March 2018. During his career, he published two influential books and over 160 scientific papers in peer-reviewed journals, the vast majority of which focused on the functional basis of drought tolerance in cereals as related to proxy traits involved in the adaptive response to water deficit. In a number of thought-provoking and at the time controversial manuscripts (e.g. Blum, 2005, 2009, 2016), he challenged some of the commonly held beliefs concerning drought resistance in crops.

Abraham pioneered and championed the study of traits and proxies (e.g. cell membrane stability, canopy temperature, waxiness, osmotic adjustment, ABA accumulation, stem reserve mobilization) able to enhance our understanding of crop plasticity under environmental constraints while providing information for predicting yield under such conditions. Amongst adaptive proxies, he was a strong advocate for osmotic adjustment (Blum, 2017) and canopy temperature (Blum *et al.*, 1982).

He was also a strong advocate of a multidiciplinary approach for investigating and enhancing drought resistance in crops. In this, his foremost and greatly appreciated contributions to those engaged in drought-related research were management of the www.plantstress.com website and organization of the InterDrought congress series, which he chaired twice, in 2005 (Rome) and 2009 (Shanghai). The InterDrought V conference (Hyderabad, 2017) community sent their good wishes to Abraham during his illness, completely covering a poster with messages of support.

We and the entire InterDrought community will miss Abraham greatly for his tremendous enthusiasm, optimism and endless dedication; his vast knowledge and contributions, and as a dear friend, teacher and colleague. Farewell Abraham.

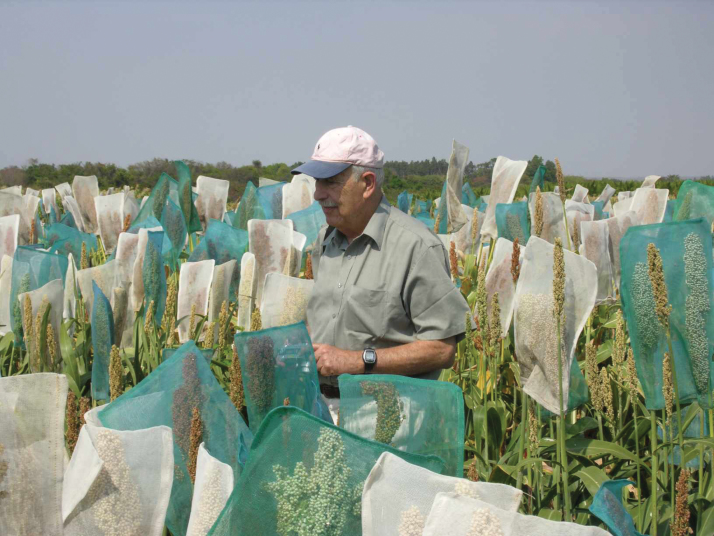

Today there is also a recognition of the diversity of drought scenarios in each region of the world in current and future climates, even in a single field over different years, and the importance of fitting plant phenology and traits to the most likely scenarios in a given region (Olesen *et al.*, 2011; Mickelbart *et al.*, 2015; Chenu *et al.*, 2018). This raises the necessity of adopting probabilistic approaches to drought tolerance based on both crop modelling and genomic prediction in identifying where and when any combination of alleles or traits are beneficial in specific drought scenarios (Ewert *et al.*, 2015; Tardieu *et al.*, 2018). Furthermore, the importance attached to modelling approaches has increased, together with an improved ability of the scientific community to more-accurately measure and forecast environmental conditions, and to simulate the behaviour of genotypes in a range of environmental conditions (Chenu *et al.*, 2018), including those associated with climate change.

## Physiological and molecular basis of drought tolerance in plants

Standard terminology is now accepted for plant water status, following Hsiao (1973), who defined ‘hydrated’, ‘mild stress’, ‘moderate stress’, ‘severe stress’ and ‘desiccation’ based on the timing and severity of water deficit. In this issue, Zhang and Bartels (2018) distinguish dehydration and desiccation tolerances, an essential distinction for phenotyping and interpreting survival and recovery (Blum and Tuberosa, 2018). These processes are achieved through multiple interactions involving stomatal conductance, carotenoid degradation and anthocyanin accumulation along with ABA and cytokinin accumulation, the intervention of osmoprotectants (e.g. sucrose, glycine, proline) and ROS-scavenging enzymes.

Turner (2018) presents 40 years of research on the beneficial role of osmotic adjustment on turgor maintenance in drought-prone environments. The genetic variations and breeding of turgor maintenance for crops resilience to water-limited environments is discussed, including examples of drought-tolerant wheat genotypes that control osmoregulation in both leaves and pollen through the expression of the *OR* gene.

A crop canopy is formed of individual plants which, although they share a common genome, have markedly different features. Borrás and Vitantonio-Mazzini (2018) analyse the role of plant-to-plant variability for plant development and ear growth, in particular via measured genetic progress over past decades. This progress is in part determined by (i) the ability of plants to produce high individual yield at high densities while maintaining sufficient uniformity between plants and (ii) the rate of silk extrusion for a given ear or plant biomass.

Failure of fertilization and arrested ovary growth can cause ovary/grain abortion, often considered to be a consequence of the availability of sucrose to ovaries (Boyer and McLaughlin, 2007; Ruan *et al.*, 2010). Here, Turc and Tardieu (2018) propose another view, that abortion under progressive water deficit is essentially due to developmental processes as affected by water deficit. This is the case in maize, pea and sunflower, with a cessation of growth of the youngest reproductive organs that occurs earlier under water deficit than in well-watered conditions, due to the effect of drought on the respective timing of the development of ovary cohorts and the signalling between oldest and youngest reproductive organs.

Drought and heat episodes are often synchronous so a combined tolerance is essential, although the genetic architectures of tolerances to drought and high temperatures differ appreciably (Tricker *et al.*, 2018). After reviewing traits with comparative advantages, these authors conclude that the maintenance of plant water status is essential for tolerance to both stresses, via fine tuning of gas exchange and plant hydraulic conductance, including the adaptive response of root systems, together with more classically described traits such as cell protection at high temperatures. This involves improving several plant plasticity traits at a time, a difficult task that can be envisaged by combining phenomics, quantitative genetics, QTL cloning and genome editing (Salvi and Tuberosa, 2015).

## Root system traits

Plant roots release a broad variety of chemical compounds to attract useful microorganisms in the rhizosphere which in turn influence plant health and growth (Huang *et al.*, 2014). Ahmed *et al.* (2018) present the root architectural and anatomical traits, together with rhizosphere traits, that affect plant water uptake. The hydraulic continuity of the rhizosphere is studied using a physical approach, and its consequences on the growth, transpiration and yield of the crop canopy are discussed.

The traits and QTLs associated with root-system architecture can have markedly different effects depending on environmental scenarios (Varshney *et al.*, 2014). A view on root system architecture in response to water deficit in legumes is presented by Ye *et al.* (2018). It brings together genetic and genomics approaches for analysing quantitative trait loci (QTLs) associated with root system architecture and the beneficial root traits that can accelerate the genetic improvement of yield under water deficit. There are already several studies where introgression of root traits has been successful in enhancing crop productivity (Varshney *et al.*, 2016).

Lynch (2018) also addresses this question and proposes that the high investment in root systems that was favoured by natural selection for crop ancestors that suffered multiple stresses and intense competition may no longer be useful in agricultural high-input agrosystems. More parsimonious root systems centred on water capture are desirable, via reduced root branching and a root anatomy that decreases the root carbon cost. Functional–structural models capable of stimulating the dynamics of root–soil interactions allow the value of these traits in different agrosystems to be evaluated. In particular, parsimonious root systems are probably less useful in low-input fields characterized by multiple stress-related cues in addition to water deficit.

## Interdisciplinary approaches

Although the concept of converging or integrating distinct disciplines is not new in plant research, it remains challenging (see Mir *et al.*, 2012). Convergence not only concerns the small area of intersection between different disciplines, but also new approaches that represent the merging and integration of different technologies, disciplines and modelling.

Chenu *et al.* (2018) integrate modelling and phenomic approaches for addressing complex traits in cereals and cover advances in cereal genomics using integrated approach including the identification of a number of significant QTLs for transpiration efficiency (biomass produced for unit of water used) in sorghum. Despite of progress in genetics, genomics and phenotyping, trait selection in breeding is limited by our ability to understand interactions within the plant and with the environment and to identify traits of most relevance for the target population of environments. Studying and extending integrated approaches via modelling can capitalize on insights gained by the drought community and elsewhere.

Genomics is generating new tools, such as functional molecular markers and informatics, as well as new knowledge about statistics and inheritance phenomena that could increase the efficiency and accuracy of crop improvement (Varshney *et al.*, 2005). Here, one review on integrating genomics, phenomics, systems modeling and agronomy for enhancing genetic gains in legumes emphasizes the role of selection intensity, generation interval and improved operational efficiencies in breeding (Varshney *et al.*, 2018). It also addresses increase in profitability of farming and availability of affordable notorious food with enhanced genetic gains in terms of not only productivity but also nutritional and market traits.

## Conclusion

The range of topics covered in this special issue should help in the integration of disciplines needed to support crop improvement and the design of novel cropping systems allowing better yields of drought-stressed plants. We hope it will provide new inspiration and creative opportunities to address the challenges posed by climate change, and anticipate convergence to continue in coming years.
